# Polysplenia syndrome with complex heart disease and jejunal atresia with malrotation in neonate: A case report

**DOI:** 10.1002/ccr3.2768

**Published:** 2020-02-28

**Authors:** Roya Arif Huseynova, Latifa A. Bin Mahmoud, Adli Abdelrahim, Majeed A. Alroiedy, Ogtay Huseynov

**Affiliations:** ^1^ Department of Neonatal Intensive Care Unit King Saud Medical City Riaydh Saudi Arabia; ^2^ King Saud Medical City Riaydh Saudi Arabia; ^3^ Azerbaijan Medical University Baku Azerbaijan

**Keywords:** heterotaxy, newborn, polysplenia, right isomerism

## Abstract

Polysplenia is heterotaxy syndrome or bilateral left‐sidedness. We report a case of polysplenia syndrome in order to draw attention to this rare syndrome that must be excluded in an infant presenting with congenital heart disease and intestinal malformations.

## Graphical abstract

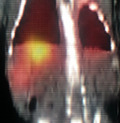

## 1 INTRODUCTION

Heterotaxy syndrome is defined as an abnormal morphology and position of the thoracoabdominal organs that do not coexist with the normal arrangement of organs with usual left‐right asymmetry (situs solitus) or reversed/mirrored arrangement of the abdominal and thoracic organs (situs inversus).^1^, ^2^

An incidence of heterotaxy syndrome is 1‐1.5/10 000 live births with a high mortality rate.^3^

Patients with right isomerism (asplenia) have a higher incidence of univentricular circulation, complete atrioventricular septal defect, pulmonary atresia, and total anomalous pulmonary venous return compared to patients with left isomerism (polysplenia). Long‐term outcome of heterotaxy syndrome determined by the severity of the cardiac anomalies.^4^

There is a high association of congenital gastrointestinal abnormalities, such as varying degrees of malrotation of the bowel, biliary atresia, volvulus, and splenic anomalies that significantly affect the long‐term survival in infants with the heterotaxy syndrome.

The aim of this report is to highlight the importance of computed tomography abdomen for detection of the spleen when the exact presence or absence of the spleen could not be detected by ultrasound and exploratory laparotomy.

## 2 CASE REPORT

A 2670‐gram male boy was a product of consanguineous marriage born to a 25‐year‐old gravida 4, para 3 mother at 40 weeks of gestation by cesarean section due to decreased fetal movement and late deceleration CTG findings.

There was no history of chronic illness or drug intake and no radiation exposure.

Baby delivered with Apgar scores 5; 7 and 7 on 1; 5 and 10 minutes, respectively.

Positive pressure ventilation was initiated due to mild respiratory distress. Venous cord gases were within the normal range. The baby shifted to the neonatal intensive care unit where was intubated due to worsening respiratory distress and started on conventional ventilation.

On examination, there were no apparent dysmorphic features.

To achieve oxygen saturation, 95% baby was needed FIO2 40%. There was mild intercostal retraction and tachypnea with respiratory rate range around 70 breath per min.

The abdomen was significantly distended with bluish discoloration and tense.

Chest radiography showed bilateral hazy lung appearance.

**Figure 1:**
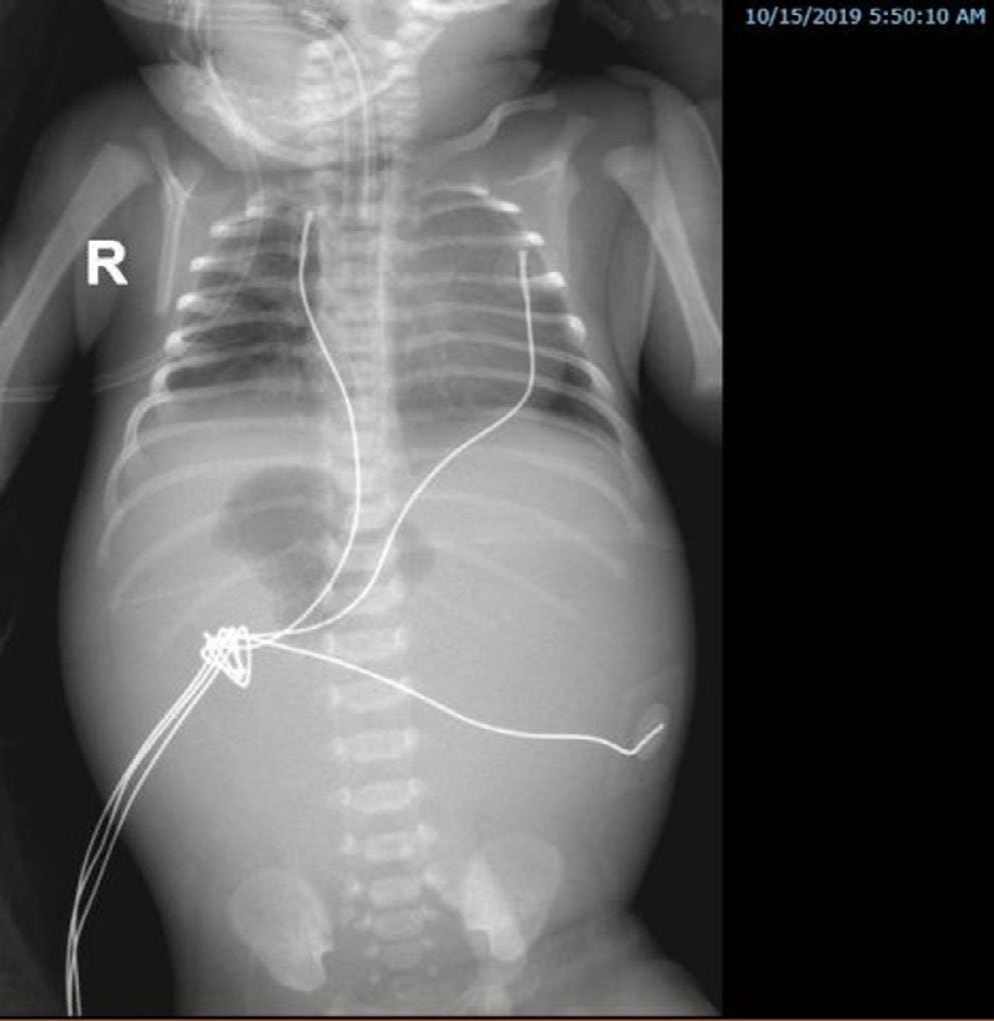
Abdominal radiography revealed a central gastric bubble with the displacement of the orogastric tube to the right side with no visible bowel pattern

The abdominal radiography revealed a central gastric bubble with the displacement of the orogastric tube to the right side with no visible bowel pattern (Figure 1).

**Figure 2:**
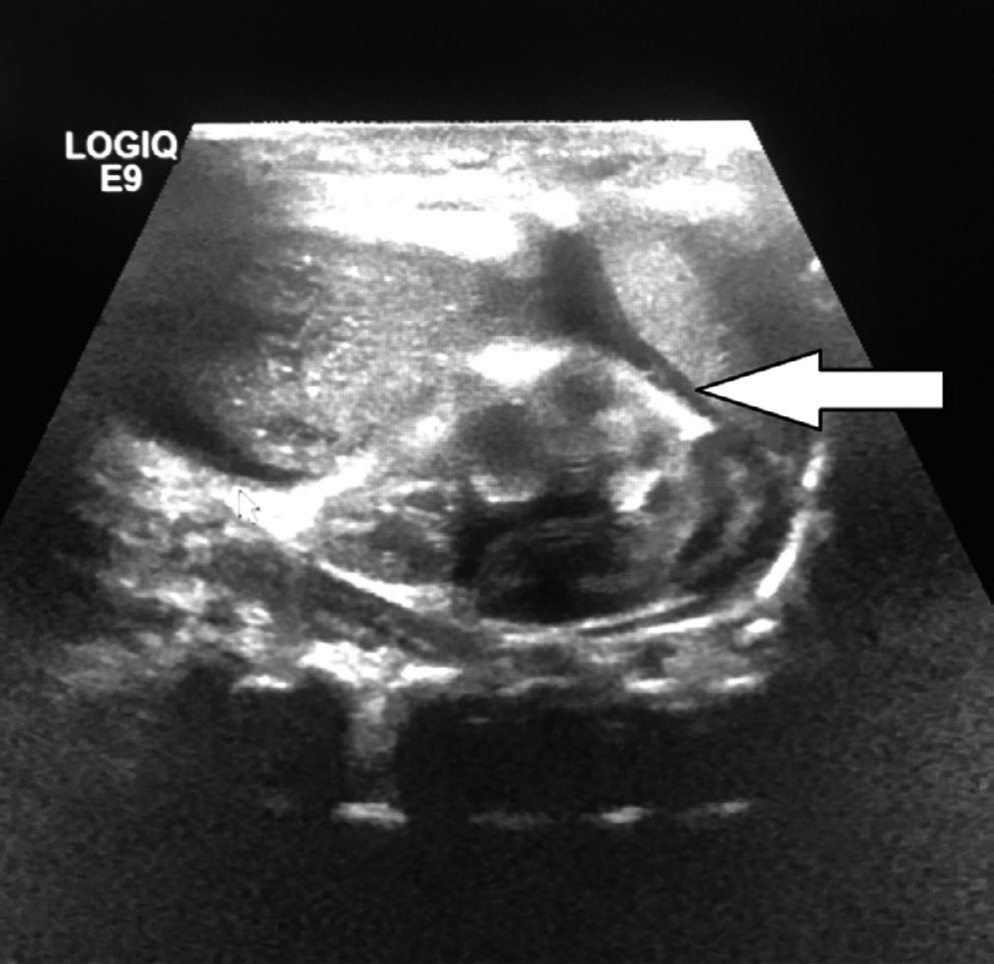
Ultrasound abdomen showed huge distended stomach with displaced liver. Spleen could not be visualized

Ultrasound abdomen showed a huge distended stomach with the displaced liver. Spleen could not be visualized (Figure 2).

**Figure 3:**
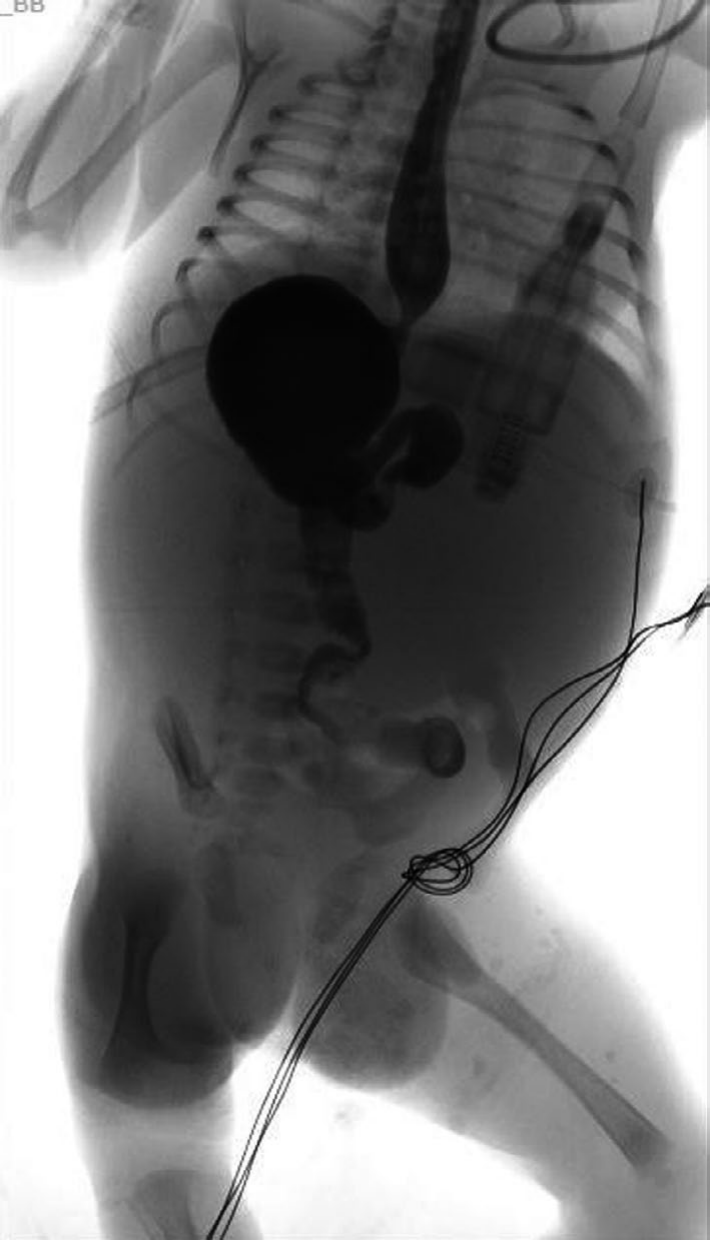
Upper gastrointestinal contrast study revealed incomplete gastric vovulus and malrotation of C‐loop of the duodenum

Upper gastrointestinal contrast study findings revealed incomplete gastric volvulus and malrotation of C‐loop of the duodenum but no obstruction (Figure 3).

Echocardiography showed hypoplastic left heart syndrome, mitral atresia, large ventricular septal defect (VSD), and interrupted aortic arch. Prostaglandin E1 continuous infusion was started.

After summarizing all available data, we considered that most probably the patient has heterotaxy right isomerism (asplenia), complex congenital heart disease, and malrotation.

Laparotomy performed on the 1st day of life showed malrotation with multiple jejunal atresia (sausage bowel), nonretracting dusky bowel with short mesentery, abnormal central position of the liver, and no spleen could be detected during the surgical procedure. The jejune‐jejunal anastomosis was performed after resection of dead bowel with preserved 35 cm of bowel remaining from the duodenojejunal junction and 35 cm from the distal part.

A peripheral blood smear was undertaken to check for the presence of Howell‐Jolly bodies to ascertain that there is no splenic function but the result did not demonstrate Howell‐Jolly bodies.

**Figure 4:**
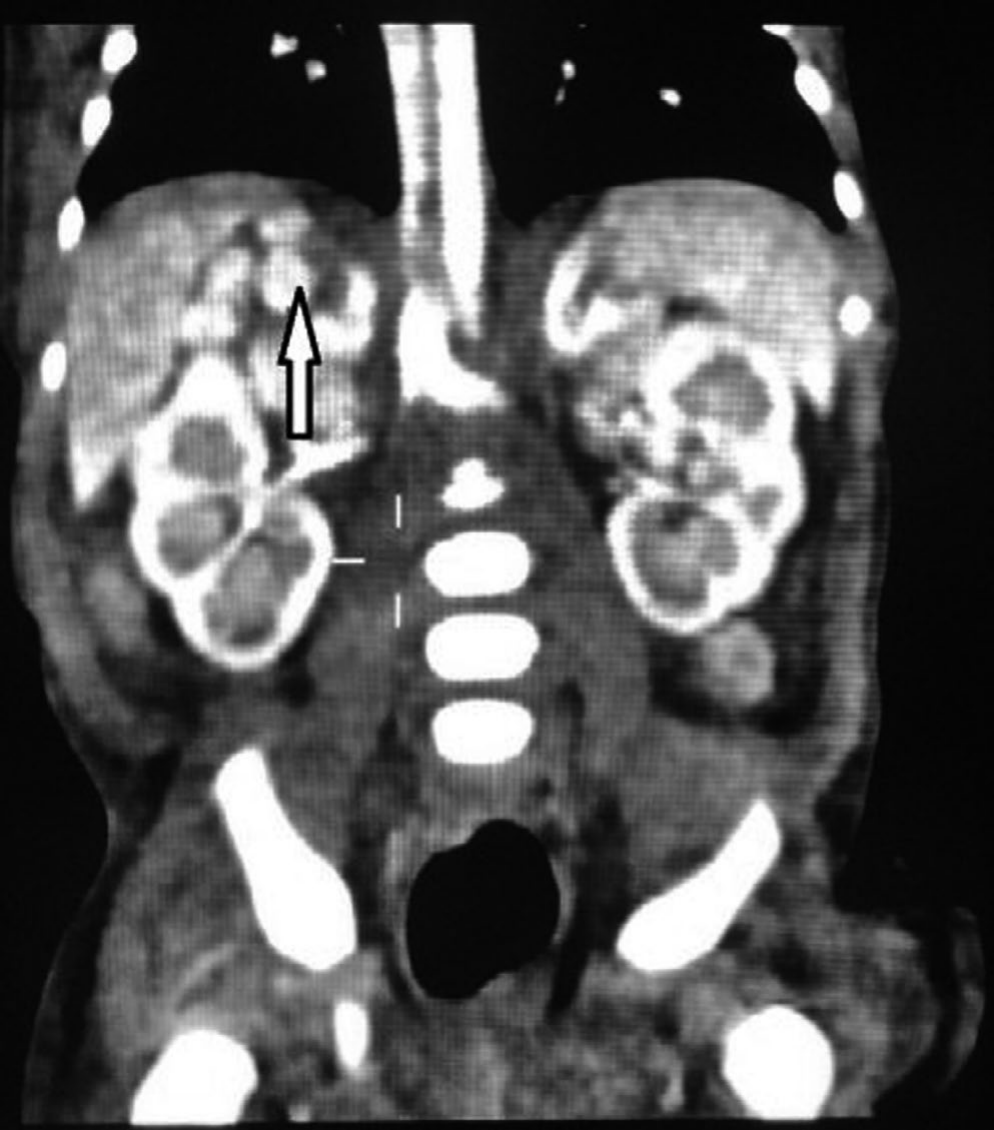
Computed tomography abdomen reported the presence of the spleen in form of 5 well‐defined small cysts like appearance in the right upper quadrant suggestive of polysplenia syndrome

For confirmation of the diagnosis of asplenia, a computed tomography abdomen was requested that reported the presence of the spleen in form of five well‐defined small cysts like appearance located in the right upper quadrant lateral to the right suprarenal gland, below and medial to the right hepatic lobe suggestive of polysplenia syndrome. Hepatic parenchyma was homogeneously enhanced and the liver enlarged, measuring 9.4 cm in length. The kidneys and pancreas were normal. Bilateral slightly prominent adrenal glands with no focal lesion were reported. The intrahepatic portion of inferior vena cava was visualized and normal. The visualized lung bases and bony skeleton are grossly unremarkable (Figure 4).

**Figure 5:**
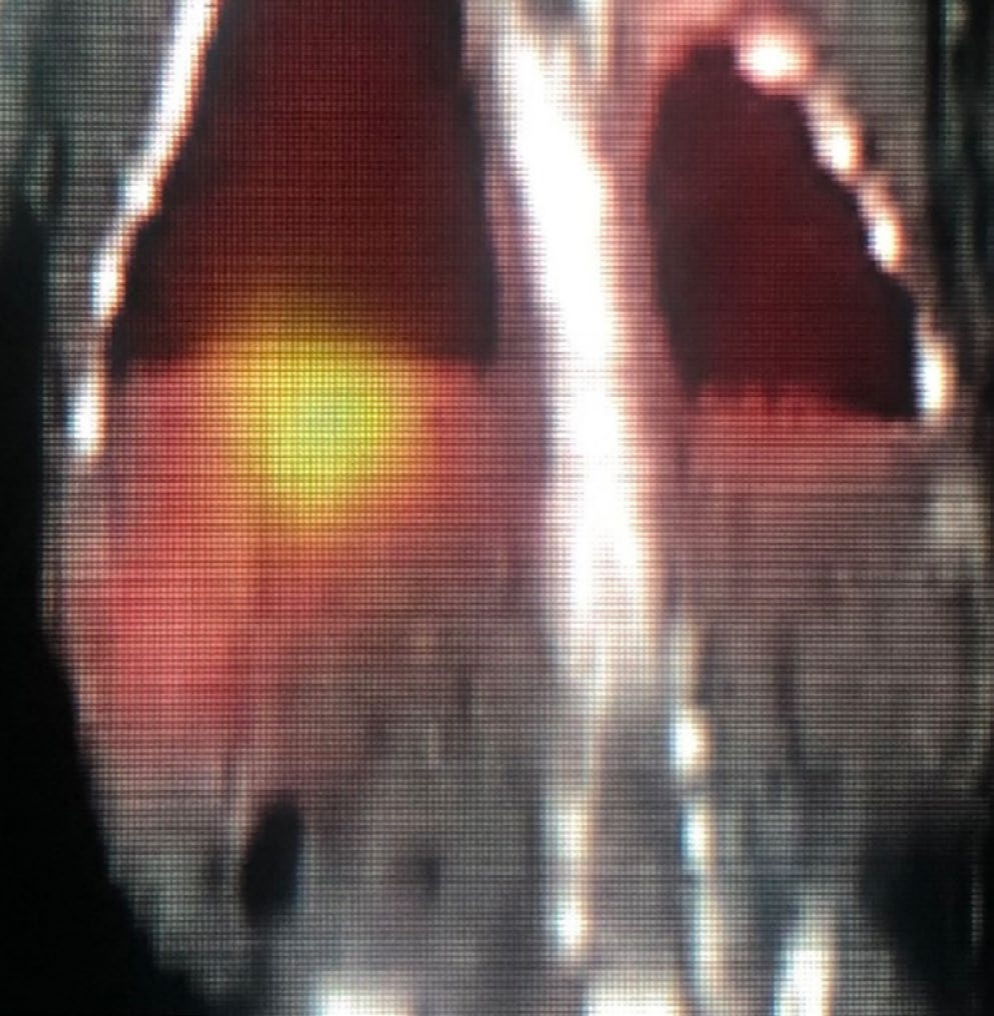
Standard spleen scan using 99m Tc‐Sulfur‐colloid showed evidence of focal area of intense activity in the right upper quadrant of the abdomen, highly suggestive of polysplenia

Standard spleen scan using 99mTc‐Sulfur‐colloid showed evidence of focal area of intense activity in the right upper quadrant of the abdomen, highly suggestive of polysplenia with adequate functional status (Figure 5).

The baby was gradually weaned from ventilatory support and extubated day 7 of life to nasal cannulae and then to room air.

Feeding was started that gradually increased until he received full maintenance feeds.

The case was referred to the higher cardiac center for cardiac defect repair but rejected due to poor outcome of repair hypoplastic left heart syndrome and palliative care was applied.

## 3 DISCUSSION

In the index case, we found polysplenia with five splenules on the right side with complex heart disease and malrotation with jejunal atresia, a combination rarely reported.

The case of situs inversus abdominous and type‐I jejunal atresia was described by Rasool and Mirza,^5^ where polysplenia was detected during laparotomy in form of seven spleens located behind and around of the stomach, with no structural cardiac anomalies. In our case, it was five spleens, that were not revealed during laparotomy due to abnormal position in the right upper quadrant and complex heart disease that is a rare combination that was not reported before.

Another recent case report published by Chinya et al^6^ described a patient with findings of complex jejunal atresia and polysplenia (three spleens) with the liver located predominantly on the left side of abdomen and levocardia with ostium secundum atrial septal defect. Polysplenia syndrome was diagnosed by abdominal ultrasound that was failed in our case.

Another case of polysplenia reported by Carcio‐Rodriguez et al^7^ was diagnosed to have 13q deletion. In addition to polysplenia and hypoplastic left heart syndrome, it also involved diaphragmatic hernia, right pulmonary sequestration, and pancreatic agenesis. All of which were intact in our patient.

Patients with polysplenia usually have less incidence of congenital heart disease with late‐onset of the serious cardiac manifestations that leads to late detection and diagnosis compared with asplenic patients.^8^

The index case supports that polysplenia can be associated with complex heart disease and significant cardiac manifestation from the first day of life that required immediate management with prostaglandin infusion.

Our case with the rare combination of polysplenia, jejunal atresia, and malrotation was complicated by hypoplastic heart syndrome that associated with a poor prognosis due to the high mortality rate.

Escobar et al^9^ in their studies concluded that the 5‐year survival rate was 86% for polysplenia syndrome compared to 53% for asplenia syndrome where noncardiac anomalies and pulmonary vein stenosis were predictors for death while in the polysplenia syndrome, the presence of univentricular circulation and left ventricular circulation were predictors for the poor outcome.

Diagnosis of heterotaxy often suspected based on radiology and ultrasonography finding of the abdominal structures that can be presented as midline‐located heart and liver, midline or right‐sided stomach. However, in some cases with heterotaxy, these findings may not be clearly revealed. Also, ultrasound is useful, but an operator‐dependent tool, and can be missed if the operator fails to recognize the anatomic relationships that occur from abnormal laterality.

For this purpose, computed tomography and magnetic resonance imaging are very useful in the evaluation of the cases with different viscerovascular abnormalities.

In index case, finding from computed tomography abdomen with contrast was diagnostic of polysplenia that was not confirmed by ultrasound of the abdomen. The definitive confirmation of polysplenia was made by a radiocolloid scan using 99mTc‐Sulfur‐colloid that was taken by the ectopic splenic tissue.

## 4 CONCLUSION

We report the rare case of polysplenia, jejunal atresia with malrotation that carries a high level of mortality if complicated with complex heart disease.

Clinical diagnosis of polysplenia can be challenging because of the difficulty detection of the spleen by abdominal ultrasound and during laparotomy due to the abnormal location. This may suggest the necessity of the CT scan of the abdomen as additional important tool for detection, location, and numbers of spleens and also the determination of possibility of other anomalies in the case of heterotaxy syndrome.

## CONFLICT OF INTEREST

None declared.

Informed Consent: Informed consent was obtained from parents for reporting this case.

## AUTHOR CONTRIBUTION

RAH: wrote the clinical report, collected and analyzed the data. All authors participated in the drafting and critically revising the manuscript. All authors approved the manuscript as submitted.
